# Keeping It Simple: A Case Report on Atomized Intranasal 1% Lidocaine as an Effective Treatment for Pain Crisis Due to Trigeminal Neuralgia

**DOI:** 10.7759/cureus.97652

**Published:** 2025-11-24

**Authors:** Drake D Dixon, Kevin Sze, Jennifer H Elfman, Shayne Gue

**Affiliations:** 1 Emergency Medicine, University of Central Florida (UCF) Hospital Corporation of America (HCA) Florida Osceola Hospital, Kissimmee, USA; 2 Pharmacy, Hospital Corporation of America (HCA) Florida Osceola Hospital, Kissimmee, USA; 3 Medical Education, University of Central Florida College of Medicine, Orlando, USA; 4 Emergency Medicine, BayCare Health System, St. Joseph's Hospital, Tampa, USA

**Keywords:** chronic pain, emergency medicine, intranasal, lidocaine, trigeminal neuralgia

## Abstract

Trigeminal neuralgia (TN) is a chronic pain disorder characterized by recurrent attacks of sudden facial pain. Unfortunately, there are few options to adequately prevent attacks of TN and even fewer options for rescue therapy during acute episodes. Successful treatment of TN pain with the readily available 1% formulation, typically used for local anesthesia, has not yet been demonstrated. We present a case report of a 37-year-old male patient who presented to the emergency department with severe, debilitating TN pain and achieved significant relief within 20 minutes of intranasal (IN) atomized 1% lidocaine.

## Introduction

Trigeminal neuralgia (TN) is defined by recurrent, short-lived episodes of unilateral electric shock-like pain in the distribution of the trigeminal nerve [1]. The pain is usually made worse with very mild stimuli such as light touch, eating, brushing the teeth, or talking [1]. These activities are essential to daily life; therefore, it is paramount to control this pain in the acute setting and beyond. Most cases of TN are caused by compression of the trigeminal nerve root [2]. The annual incidence of TN is reported as 4.7-28.9 per 100,000 person-years [3]. First-line treatments for chronic pain management require time to achieve therapeutic efficacy. Consequently, an effective and dependable approach to short-term pain relief is essential.

The 2008 guidelines from the American Academy of Neurology and European Federation of Neurological Sciences recommend carbamazepine (level A recommendation) or oxcarbazepine (level B) as the first-line therapy for TN [4]. Carbamazepine works by “inhibiting voltage-gated sodium channels, thereby reducing the excitability of neural membranes” [2]. The starting dose of carbamazepine is typically 100 to 200 mg twice daily. However, titration is usually needed up to 600 to 800 mg daily for adequate relief. This may take days to weeks in order to minimize side effects. A 2005 Cochrane review found that the number needed to treat for the effectiveness of carbamazepine was 1.8 (95% CI 1.4-2.8) [5]. The number needed to harm for severe adverse events is 24 [5]. Around 27% of patients may have to stop or reduce the dose due to poor tolerability with adverse effects such as nausea, vomiting, diarrhea, rash, pruritus, drowsiness, dizziness, blurred vision, lethargy, and headache [3]. Alternatively, oxcarbazepine, the keto derivative of carbamazepine, can be used, but it also takes significant time to achieve satisfactory pain relief. The typical starting dose of oxcarbazepine is 150 mg twice daily and can be titrated up to a maximum of 1800 mg per day. Oxcarbazepine has also been cited to have better tolerability and decreased risk of drug interactions, as it is processed by the liver cytochrome system [6]. However, the same issue arises: it takes substantial time to reach significant pain relief.

Recommended second-line therapies include baclofen and lamotrigine, followed by third-line therapies that include levetiracetam, gabapentin, pregabalin, topiramate, and botulinum toxin injections [2]. Unfortunately, these medications also take significant time to provide adequate pain relief. Botulinum toxin injections may be beneficial, but data are limited, and the toxin is not readily available or routinely administered in the ED.

The majority of the TN treatment options reviewed above are aimed at reducing the severity and frequency of attacks, not to provide pain relief during an acute episode. These medications require titration over time, and therefore, they are ineffective as acute analgesics. Rescue therapies include intravenous (IV) or intranasal (IN) lidocaine, IV phenytoin or fosphenytoin, or subcutaneous sumatriptan. Lidocaine IV at 5 mg/kg over one hour showed some benefit in a placebo-controlled crossover trial of 20 patients [7]. However, administration of IV lidocaine requires continuous telemetry and frequent blood pressure checks. IN or intraoral lidocaine has been reported as effective in a retrospective study of 152 patients in China [8]. In that study, clinicians administered 2.4 percent lidocaine aerosol (32 mg per dose) and reported at least 50% improvement in pain at 15 and 30 minutes in 78 and 70 percent of patients, respectively. Another study also reported similar relief using IN lidocaine 8% (16 mg per dose) [9]. One common limitation of these studies is that aerosolized lidocaine and 8% lidocaine are not readily available in most EDs. Here, we present a case study of a patient who achieved significant relief from severe TN pain using easily obtainable 1% lidocaine.

## Case presentation

A 37-year-old man with a past medical history of TN presented to our ED with severe right-sided temporomandibular joint (TMJ) pain that radiated into his forehead and jaw. The patient had been diagnosed with TN five days prior to evaluation in our ED by his primary care physician. At that time, the patient was started on prednisone 50 mg daily, carbamazepine 200 mg daily, and hydrocodone-acetaminophen 10/325 mg daily. Despite the initiation of this treatment, the patient presented to our ED with intractable pain. He described the pain as an “electric, shooting” sensation that started in the right TMJ region and spread to the forehead and jaw. The pain worsened with any movement of the jaw. As a result, the patient would not talk, eat, or brush his teeth due to the pain. During the initial assessment, the patient frequently turned to his phone to text what he wanted to say due to the pain associated with talking. He reported the attacks come in clusters and that his current pain level was 10 on a 1 to 10 numerical rating scale (NRS). The patient denied any headaches, vision changes, numbness, focal weakness, throat pain, chest pain, shortness of breath, nausea, vomiting, fevers, chills, cough, abdominal pain, flank pain, or dysuria.

On physical examination, the patient exhibited frequent spasms of the musculature of the right side of his face that would coincide with pain attacks. The muscle spasms involved the right eye, face, and lips. The paroxysms of pain were very brief, occurring for a few seconds at a time. The patient did not have any motor or sensory deficits of the face or any other part of the body. His physical exam was otherwise unremarkable. Initial diagnostic evaluation in the ED included laboratory analysis with a complete blood count and complete metabolic profile, which were unremarkable. We also obtained computed tomography angiography (CTA) of the head and neck, which revealed no stenosis and no large vessel occlusion; however, it was significant for a tortuous vertebrobasilar system that likely did not impinge on the trigeminal root entry zones. For initial management, the patient was given 4 mg of IV morphine, 10 mg of IV dexamethasone, and 15 mg of IV ketorolac and reported no relief of symptoms. Given the intractable pain despite multiple analgesics, we opted to trial atomized lidocaine, to which the patient agreed. We administered 1 mL of 1% IN atomized lidocaine into each nostril (10 mg per naris) for a total of 20 mg, after which the patient reported significant relief of symptoms. After 20 minutes, the patient reported a significant subjective decrease in pain, rating his pain a 4 to 5 out of 10, down from an initial pain score of 10. This represented a 50 to 60% decrease in pain. He was extremely satisfied with the treatment provided and was subsequently admitted to the internal medicine service for further evaluation and management of his condition with neurology consultation.

**Figure 1:**
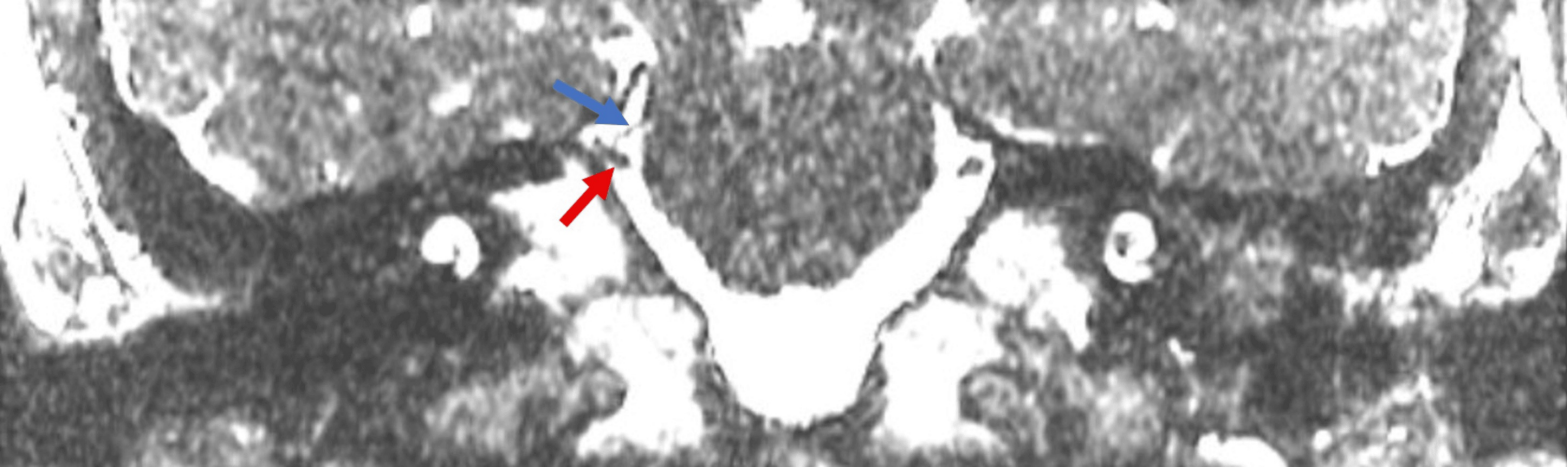
A coronal slice of an MRI brain using the FIESTA protocol. The right superior cerebellar artery (blue arrow) abuts the dorsal aspect of the proximal right trigeminal nerve (red arrow). FIESTA: Fast Imaging Employing Steady-State Acquisition

While inpatient, the patient underwent magnetic resonance imaging (MRI) of the brain, along with magnetic resonance angiography (MRA) of the head and neck. These studies revealed the right superior cerebellar artery abutting the dorsal aspect of the proximal right trigeminal nerve, which is hypothesized to have been contributing to his symptoms (Figure 1).

During hospitalization, the patient received 2 mL of 1% atomized lidocaine every six hours while oxcarbazepine was being titrated up to effect. He was discharged home on hospital day four with a prescription for twice-daily oxcarbazepine 300 mg and 1% lidocaine to be administered intranasally for breakthrough pain. He was also instructed to follow up with neurology on an outpatient basis within one to two weeks.

## Discussion

Carbamazepine and oxcarbazepine received level A and level B recommendations, respectively, from the American Academy of Neurology as first-line therapies for TN due to multiple studies demonstrating their efficacy [4]. However, in the emergent setting, these treatments are of limited utility due to the time needed for titration, making them inadequate to provide short-term relief, leaving patients frustrated and in pain. Although carbamazepine’s plasma concentrations peak within four to five hours after administration of the immediate-release oral tablets, it may take weeks to prove effective in decreasing the frequency and severity of pain episodes [10]. Oxcarbazepine has similar pharmacokinetics, taking anywhere from 4.5 to six hours to reach maximum plasma concentrations after a single dose [11].

There have been several smaller studies investigating rescue treatments for acute TN attacks; however, none have been studied on a large scale with stringent methodology. In 2023, a retrospective study of 152 patients in China investigated the use of a 2.4% lidocaine IN/intraoral aerosolized spray, demonstrating reductions in pain at 15 and 30 minutes of 77.6% and 70.4% in patients, respectively [8].

In this case, we observed the effects of 1% atomized lidocaine on acute TN pain. We chose 1% lidocaine due to the ease of access and availability of this concentration of the medication in the ED. As demonstrated in the case presentation, the patient experienced significant relief after 20 minutes of administration of 1% IN atomized lidocaine, leading the neurology team to continue this method of pain relief in both the inpatient and outpatient settings.

We administered 1 ml of the readily available 1% solution of lidocaine atomized to each nostril, a total of 20 mg of lidocaine, which was well under the accepted maximum dosage of 5 mg/kg. Given the significant improvement in pain and patient satisfaction in our case, we believe IN lidocaine is a promising abortive therapy for TN in the acute setting.

## Conclusions

TN is a frustrating and difficult-to-manage chronic pain syndrome in the acute setting. Current treatment options focus on long-term reduction of the frequency and severity of pain symptoms but do not treat the acute pain episode, leaving patients frustrated with the perception that their pain was not adequately managed. Current literature regarding abortive treatment is sparse, though there have been some promising therapies. In this case study, we demonstrate the proof of concept utilizing IN atomized lidocaine with a large safety margin. We believe IN lidocaine may be a potential therapy for abortive pain management in the acute setting, but it will require further research investigating its efficacy.
